# Placebo Response and Media Attention in Randomized Clinical Trials Assessing Cannabis-Based Therapies for Pain: A Systematic Review and Meta-analysis

**DOI:** 10.1001/jamanetworkopen.2022.43848

**Published:** 2022-11-28

**Authors:** Filip Gedin, Sebastian Blomé, Moa Pontén, Maria Lalouni, Jens Fust, Andreé Raquette, Viktor Vadenmark Lundquist, William H. Thompson, Karin Jensen

**Affiliations:** 1 Department of Clinical Neuroscience, Karolinska Institutet, Stockholm, Sweden; 2 Department of Applied Information Technology, University of Gothenburg, Gothenburg, Sweden

## Abstract

**Importance:**

Persistent pain is a common and disabling health problem that is often difficult to treat. There is an increasing interest in medicinal cannabis for treatment of persistent pain; however, the limited superiority of cannabinoids over placebo in clinical trials suggests that positive expectations may contribute to the improvements.

**Objective:**

To evaluate the size of placebo responses in randomized clinical trials in which cannabinoids were compared with placebo in the treatment of pain and to correlate these responses to objective estimates of media attention.

**Data Sources:**

A systematic literature search was conducted within the MEDLINE and Embase databases. Studies published until September 2021 were considered.

**Study Selection:**

Cannabinoid studies with a double-blind, placebo-controlled design with participants 18 years or older with clinical pain of any duration were included. Studies were excluded if they treated individuals with HIV/AIDS or severe skin disorders.

**Data Extraction and Synthesis:**

The study followed the Preferred Reporting Items for Systematic Review and Meta-analyses reporting guideline. Data were extracted by independent reviewers. Quality assessment was performed using the Risk of Bias 2 tool. Attention and dissemination metrics for each trial were extracted from Altmetric and Crossref. Data were pooled and analyzed using a random-effects statistical model.

**Main Outcomes and Measures:**

Change in pain intensity from before to after treatment, measured as bias-corrected standardized mean difference (Hedges *g*).

**Results:**

Twenty studies, including 1459 individuals (mean [SD] age, 51 [7] years; age range, 33-62 years; 815 female [56%]), were included. Pain intensity was associated with a significant reduction in response to placebo, with a moderate to large effect size (mean [SE] Hedges *g*, 0.64 [0.13]; *P* < .001). Trials with low risk of bias had greater placebo responses (*q*_1_ = 5.47; *I*^2^ = 87.08; *P* = .02). The amount of media attention and dissemination linked to each trial was proportionally high, with a strong positive bias, but was not associated with the clinical outcomes.

**Conclusions and Relevance:**

Placebo contributes significantly to pain reduction seen in cannabinoid clinical trials. The positive media attention and wide dissemination may uphold high expectations and shape placebo responses in future trials, which has the potential to affect the outcome of clinical trials, regulatory decisions, clinical practice, and ultimately patient access to cannabinoids for pain relief.

## Key Points

**Question:**

What is the size of the placebo response in cannabinoid trials for clinical pain, and is the magnitude of placebo response associated with media attention on the trials?

**Findings:**

This meta-analysis of 20 studies of 1459 individuals found a significant pain reduction in response to placebo in cannabinoid randomized clinical trials. Media attention was proportionally high, with a strong positive bias, yet not associated with the clinical outcomes.

**Meaning:**

These findings suggest that placebo has a significant association with pain reduction as seen in cannabinoid clinical trials, and the positive media attention may shape placebo responses in future trials.

## Teaser

This meta-analysis evaluates the size of placebo responses in double-blind randomized clinical trials of cannabinoid treatment for pain and the association of media attention on those trials with participant responses.

## Introduction

Pain is one of the most common reasons for seeking health care, and persistent pain leads to major disability and poor quality of life.^1,2,3^ Persistent pain is the leading cause for years lived with disability^3,4^ and thereby represents a major challenge for health care systems and society as a whole.

In recent years, there has been an increasing interest in the medical properties of cannabinoids, and countries are starting to introduce cannabinoids as part of their regular health care.^5^ Medicinal cannabinoids are currently used to treat multiple pain conditions, such as back pain and cancer pain.^6^ Despite the increased demand for cannabinoids among individuals with persistent pain, the evidence is deemed low, or very low, for analgesic efficacy.^6^ Although patients improve in double-blind, placebo-controlled trials, there is limited superiority of cannabinoids over the placebo response, which suggests that the placebo response contributes considerably to the pain reduction seen in cannabinoids in clinical trials. There is ample evidence of placebo responses in treatment studies for long-term pain.^7^ Contextual factors play an important role because communication about treatments, their effects, and their adverse effects shapes expectations and placebo analgesic responses.^8^

The first aim of this systematic review and meta-analysis was to evaluate the size of placebo responses in double-blind randomized clinical trials in which cannabinoids, cannabis, and cannabis-based medicine were compared with placebo in the treatment of clinical pain. A second aim was to examine the association of the clinical outcomes with the amount of attention and engagement each trial has received in both scientific and nonscientific contexts. Placebo responses are shaped by contextual factors and are therefore susceptible to reports in the mass media and lay press.^9^

## Methods

This study was conducted in accordance with the Preferred Reporting Items for Systematic Reviews and Meta-analyses (PRISMA) reporting guideline.^10^ The study protocol was preregistered at PROSPERO (CRD42021248492), and no changes were made after the registration.

### Participants and Interventions

Studies with individuals 18 years or older with clinical pain of any duration were included. Data on race and ethnicity were not collected because only 7 of the 20 articles included in this meta-analysis provided these data. Studies that included individuals with HIV/AIDS or a severe skin disorder were excluded. Studies of all types of cannabinoids (natural or synthetic) and their placebo equivalents designed to serve as double-blind comparators were included.

### Outcomes and Design

The primary outcome was the change in self-reported pain intensity from before to after treatment. There are many ways to assess pain in clinical trials; however, this meta-analysis included only trials that measured pain intensity (as opposed to, for example, pain impact) with a self-rating scale (visual or numeric scale). The secondary outcome was the association between study effect size and Altmetric scores or Crossref citations.

### Search Strategy and Study Selection

The literature search was performed at the Karolinska Institute University Library in the MEDLINE and Embase databases. All placebo-controlled randomized clinical trials that focused on cannabinoid treatment for clinical pain were included. There were no restrictions on publication date or language (see eTable 1 in Supplement 1 for the complete search strategies). All studies published up until September 2021 (date of search) were included.

Titles and abstracts found in the literature search were first screened for eligibility by 2 independent reviewers (F.G. and M.P. or S.B.; M.L. and W.H.T.; J.F. and V.V.L.; or A.R. and M.P.), using the Rayyan web tool (Qatar Computing Research Institute). Second, 2 independent reviewers (F.G. and V.V.L. or S.B.; M.L. and J.F.; or A.R. and M.P.) screened the eligibility of the full-text articles. Any disagreements were resolved in discussion with a third reviewer (K.J.).

### Data Collection and Study Appraisal

All data regarding sample size, characteristics of the study population, outcome measurements, and results were extracted by 2 independent reviewers (F.G. and S.B.). If there were any disagreements, a third reviewer (K.J.) was consulted.

### Risk of Bias

To assess the methodologic quality of the included studies, each article was scored according to a modified version of the Risk of Bias 2 (ROB2) tool for randomized clinical trials,^11^ including 5 domains: (1) randomization, (2) deviations from the intended interventions, (3) missing outcome data, (4) measurement of the outcome, and (5) selection of the reported result. Because successful blinding of treatment type (active drug vs placebo) was of particular interest, a sixth domain was added that specified blinding. To assess blinding, we used the standardized blinding items included in the overall domain score for the ROB2 domains: deviations from the intended interventions and measurement of the outcome. The risk of bias (low, moderate, or high) was assessed by 2 independent reviewers (F.G. and V.V.L. or S.B.; M.L. and J.F.; or A.R. and M.P.), and any disagreements were solved by a third reviewer (K.J.). A study was considered to have low risk of bias if all domains were categorized as low. A study was considered to have high risk of bias if 2 or more domains had high risk or if the study had 1 domain with high risk and more than 1 domain with moderate risk of bias. Publication bias was assessed using a funnel plot.

### Altmetric Scores

Altmetric scores quantified general and social media attention linked to a specific scientific article (nonacademic impact). Furthermore, we used Crossref to quantify the number of academic citations an article received (academic impact). We obtained the Altmetric scores through Altmetric’s application programming interface (API) on February 14, 2022 (overall scores and age-adjusted percentiles), and information about media and blog posts on February 21, 2022, using Almetric’s Details Page API. The code used to obtain overall scores from the Altmetric API and Crossref scores can be found at github.^12^

To evaluate the mass media effect of the articles, we extracted the headline, summary, and website address of all blogs and mass media posts connected to each of the 20 scientific articles in this meta-analysis, using Altmetric’s Details Page API. Two researchers (F.G. and W.H.T.) independently analyzed each news item to determine whether it was positive, negative, or neutral about the effectiveness of cannabinoids for treatment of pain specifically (ie, if a news item was positive about cannabinoid effectiveness as sleep medication but neutral to treating pain, it would receive a neutral rating). The news item’s website was solicited when the headline or summary was insufficient. News items did not receive a score if they were not in English or appeared as a temporary link. Results were compared, and if consensus was not achieved, a third researcher (K.J.) was consulted. If news items included identical text (eg, when multiple local newspapers published the same article), they were still included.

We calculated the number and proportion of positive news items compared with neutral or negative. This measure was then correlated with treatment effect size in each study using Spearman correlations and shown descriptively compared with the risk of bias.

### Statistical Analysis

Comprehensive Meta-Analysis, version 3.0 (Biostat Inc) was used for management of data and to test the standardized mean differences (Hedges *g*). Effect size is commonly interpreted as small (Hedges *g* < 0.2), moderate (Hedges g of 0.2-0.8), or large (Hedges *g* > 0.8). The treatment response to placebo and cannabinoids were analyzed separately per treatment group as measured from before to after treatment. For completeness, a traditional comparison of treatment response to genuine drug vs placebo was also performed. All analyses were performed with a random-effects approach using a 2-tailed α = .05. A metaregression model was used to test whether any of the following prespecified factors would be associated with study results: participant mean age, risk of bias, blinding success, Altmetric score, Crossref, and journal impact factor. Categorical factors were analyzed as group comparisons and include financial interest (as reported by study sponsor or investigator), substance type, route of drug administration, and type of pain condition. Correlations (2-tailed) between individual factors were performed with a Spearman ρ in SPSS software, version 26 (IBM Corp).

## Results

### Included Studies

**Figure 1.**
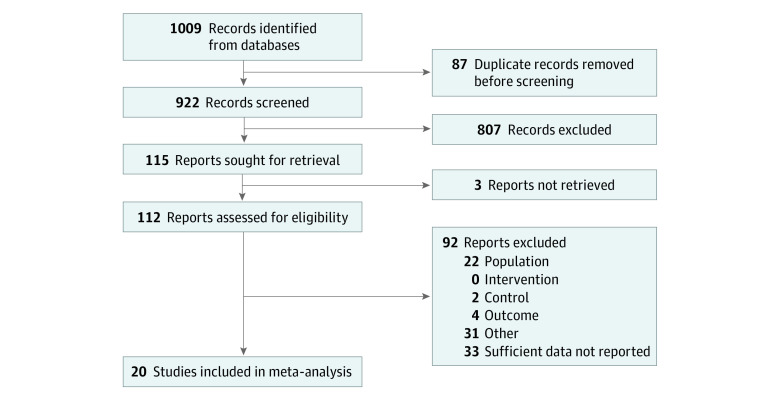
Flow Chart of Articles Included in the Meta-analysis

**Figure 2.**
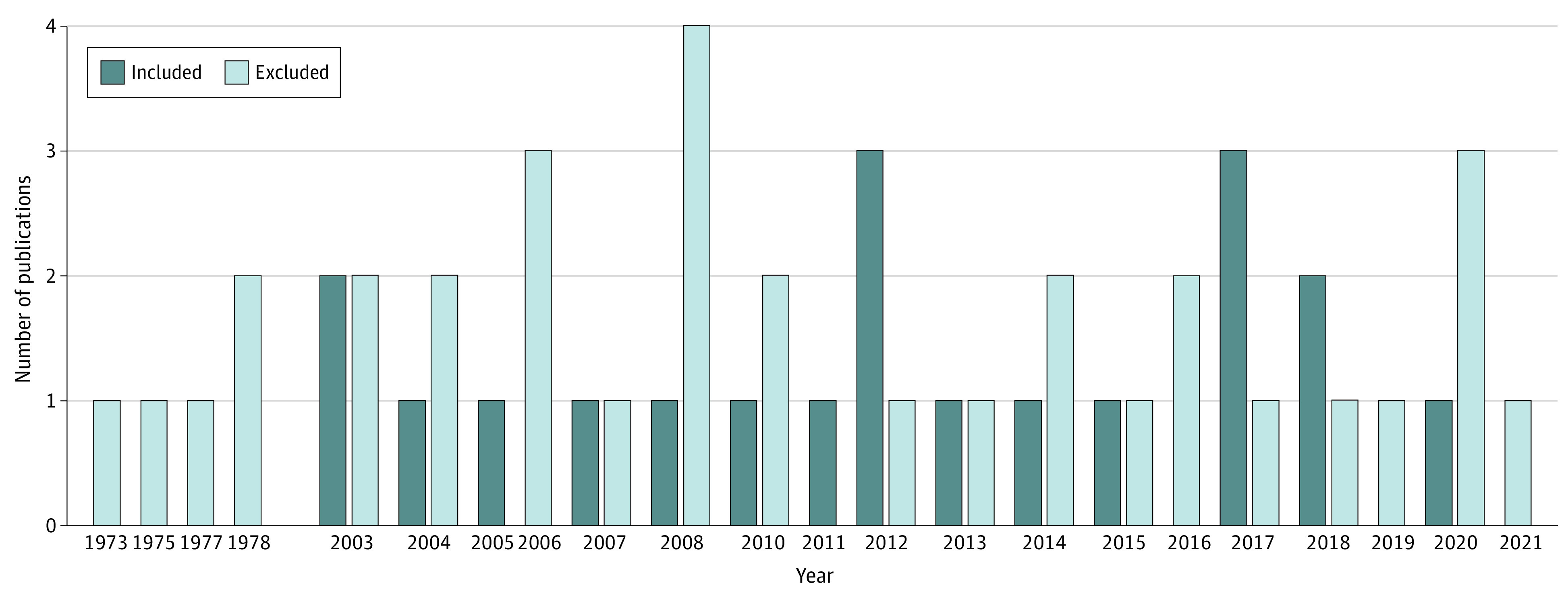
Publication Year of Articles Included in the Meta-analysis Number of publications per year for all 53 articles that met the inclusion criteria in the present meta-analysis from 1973 to 2021.

The 20 included trials included a total of 1459 individuals with pain (mean [SD] age, 51 [7] years; age range, 39-62 years; 815 female [56%] and 644 male [44%]). The literature search identified 1009 articles, of which 53 met the inclusion criteria (Figure 1). Of these, 33 studies were excluded because data were insufficient for calculation of the effect size (separate values for drug and placebo before and after treatment) and the authors did not provide data on request. Thus, a total of 20 articles were included in the meta-analysis (Figure 2).^13,14,15,16,17,18,19,20,21,22,23,24,25,26,27,28,29,30,31,32^ For a comprehensive list of the excluded articles, see eTable 2 in Supplement 1.

The included pain conditions were neuropathic pain, multiple sclerosis, and other. The treatments used in the studies were tetrahydrocannabinol and/or cannabidiol (CBD), nabilone, dronabinol, and nabiximols. Cannabidiol differs from other types of cannabinoids because it is not psychotropic. Cannabidiol was used in 3 trials.^23,24,32^ Treatment was administered as a pill, spray, oil, or smoke or vapor. Twelve of 20 studies^14,16,17,18,20,22,25,27,29,30,31,32^ included in the meta-analysis did not report any financial interest that could lead to a conflict of interest (Table).

**Table.** Study Characteristics of All Trials Included in the Meta-analysis

Source	No. of participants	Mean age, y	Female, No. %	Study duration	Substance	Type of pain	Route of administration	Crossover design	Financial interest
Berman et al,^13^ 2004	48	39	0	1.8 wk	Nabiximols	Neuropathic	Spray	Yes	Yes
Buggy et al,^14^ 2003	40	48	40 (100)	6 h	THC	Other	Pill	No	No
Chaves et al,^15^ 2020	17	52	9 (100)	8 wk	THC	Other	Oil	No	Yes
Corey-Bloom et al,^16^ 2012	30	51	19 (63)	45 min	THC	MS	Smoked	Yes	No
De Vries et al,^17^ 2017	50	52	11 (22)	12 wk	THC	Other	Pill	No	No
Issa et al,^18^ 2014	29	51	16 (53)	8 h	Dronabinol	Other	Pill	Yes	No
Langford et al,^24^ 2013	339	49	117 (68)	14 wk	THC or CBD	Multiple	Spray	No	Yes
Malik et al,^25^ 2017	13	43	11 (85)	4 wk	Dronabinol	Other	Pill	No	No
Nurmikko et al,^26^ 2007	125	53	74 (59)	5 wk	Nabiximols	Neuropathic	Spray	No	Yes
Rog et al,^23^ 2005	63	49	52 (79)	4 wk	THC or CBD	MS	Spray	No	Yes
Schimrigk et al,^19^ 2017	240	48	175 (73)	16 wk	Dronabinol	Neuropathic	Spray	No	Yes
Skrabek et al, 2008^20^	40	50	37 (93)	4 wk	Nabilone	Other	Pill	No	No
Toth et al,^21^ 2012	26	62	13 (50)	9 wk	Nabilone	Neuropathic	Pill	No	Yes
Turcott et al,^22^ 2018	33	61	26 (79)	8 wk	Nabilone	Other	Pill	No	No
Wade et al,^28^ 2003	24	48	12 (50)	6 wk	THC	Neuropathic	Spray	Yes	Yes
Wallace et al,^27^ 2015	16	57	7 (44)	4 h	THC	Neuropathic	Vaporized	Yes	No
Ware et al,^29^ 2010	23	45	12 (52)	2 wk	THC	Neuropathic	Pill	Yes	No
Weizman et al,^30^ 2018	15	33	0	4 h	THC	Neuropathic	Spray	Yes	No
Zadikoff et al,^31^ 2011	9	60	9 (100)	8 wk	Dronabinol	Neuropathic	Pill	Yes	No
Zajicek et al,^32^ 2012	279	52	175 (65)	12 wk	THC or CBD	MS	Pill	No	No

Abbreviations: CBD, cannabidiol; MS, multiple sclerosis; THC, tetrahydrocannabinol.

### Risk of Bias

Fourteen of 20 studies^13,14,16,17,18,19,23,25,26,27,29,30,31,32^ were deemed to have a moderate risk of bias, 4 studies^20,21,22,28^ to have a high risk of bias, and 2 studies^15,24^ to have a low risk of bias (eTable 3 in Supplement 1). The domain in which most studies had moderate or high risk of bias was reporting (domain 5), followed by blinding (domain 6) (eTable 3 in Supplement 1). No indication of publication bias was detected in the funnel plots (*z* = 13.04) (eFigure in Supplement 1).

### Placebo Response

**Figure 3.**
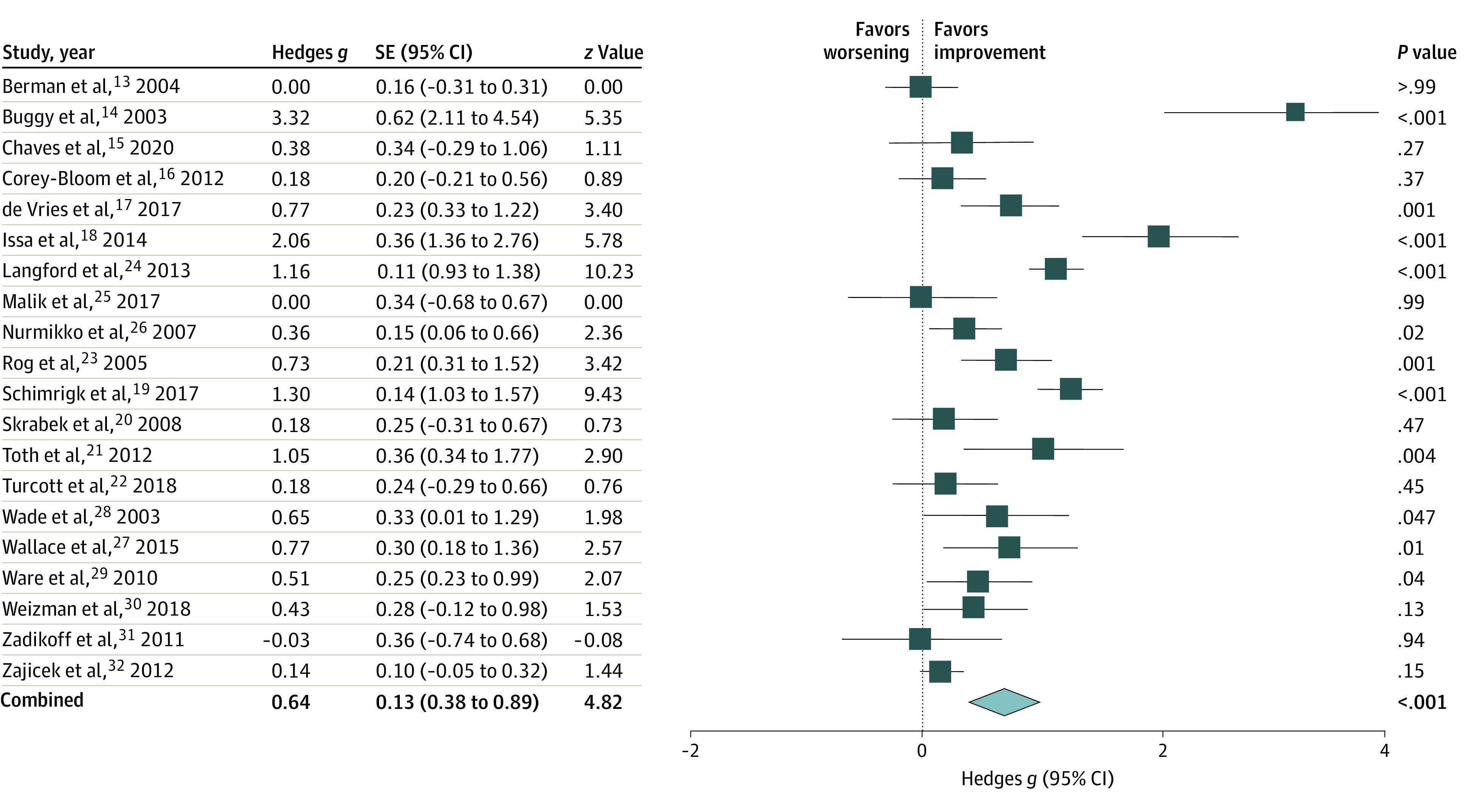
Association Between Placebo and Change in Pain Intensity Ratings The overall treatment response to placebo was statistically significant. The blue squares to the right of the midline represent improvements in pain intensity after treatment, squares on the midline represent no change, and squares to the left of the midline represent worsening of pain intensity after treatment.

Placebo cannabinoids had a statistically significant association with pain intensity, with a moderate to large effect size (mean [SE] Hedges *g*, 0.64 [0.13]; *I*^2^ = 87.08; *P* < .001). The effect size of the active drug (cannabinoids) on pain intensity was large (mean [SE] Hedges *g*, 0.95 [0.13]; *I*^2^ = 84.07; *P* < .001). However, the between-group difference for active drug and placebo was not statistically significant (*q*_1_ = 2.82; *P* = .09; Hedges *g* [cannabinoid *g* – placebo *g*] = 0.32). No substantial heterogeneity was found among the studies, as indicated by the high *I*^2^ number.^13^ For a forest plot of the improvements in the placebo group, see Figure 3.

### Moderator Analysis

A metaregression analysis revealed a significant association between risk of bias and placebo response (*q*_1_ = 5.47; *I*^2^ = 87.08; *P* = .02), with studies with a low risk of bias having higher placebo responses. The study duration varied greatly among the trials in the analysis, ranging from 45 minutes to 16 weeks. However, no association was found between study duration and the magnitude of the placebo response (*q*_1_ = 0.54; *I*^2^ = 87.08; *P* = .54). No significant results were found for any other predefined moderators, including patient age, journal impact factor, or publication year. In line with the significant association between risk of bias and placebo response, there was a significant association between blinding and placebo response (*q*_1_ = 4.26; *I*^2^ = 87.08; *P* = .04) wherein poor blinding was associated with lower placebo response. The same was not present in response to genuine treatment (*q*_1_ = 1.21; *I*^2^ = 84.07; *P* = .27).

### Group Comparisons

No significant association was found between placebo response and route of treatment administration, comparing traditional pills with more elaborate procedures, including spray, oil, smoke, and vaporized cannabis. The associations of placebo response and type of pain, presence of financial interest in a trial, type of substance, and type of pain condition were also not significant.

### Scientific Citations, Journal Impact Factor, and Altmetric Scores

**Figure 4.**
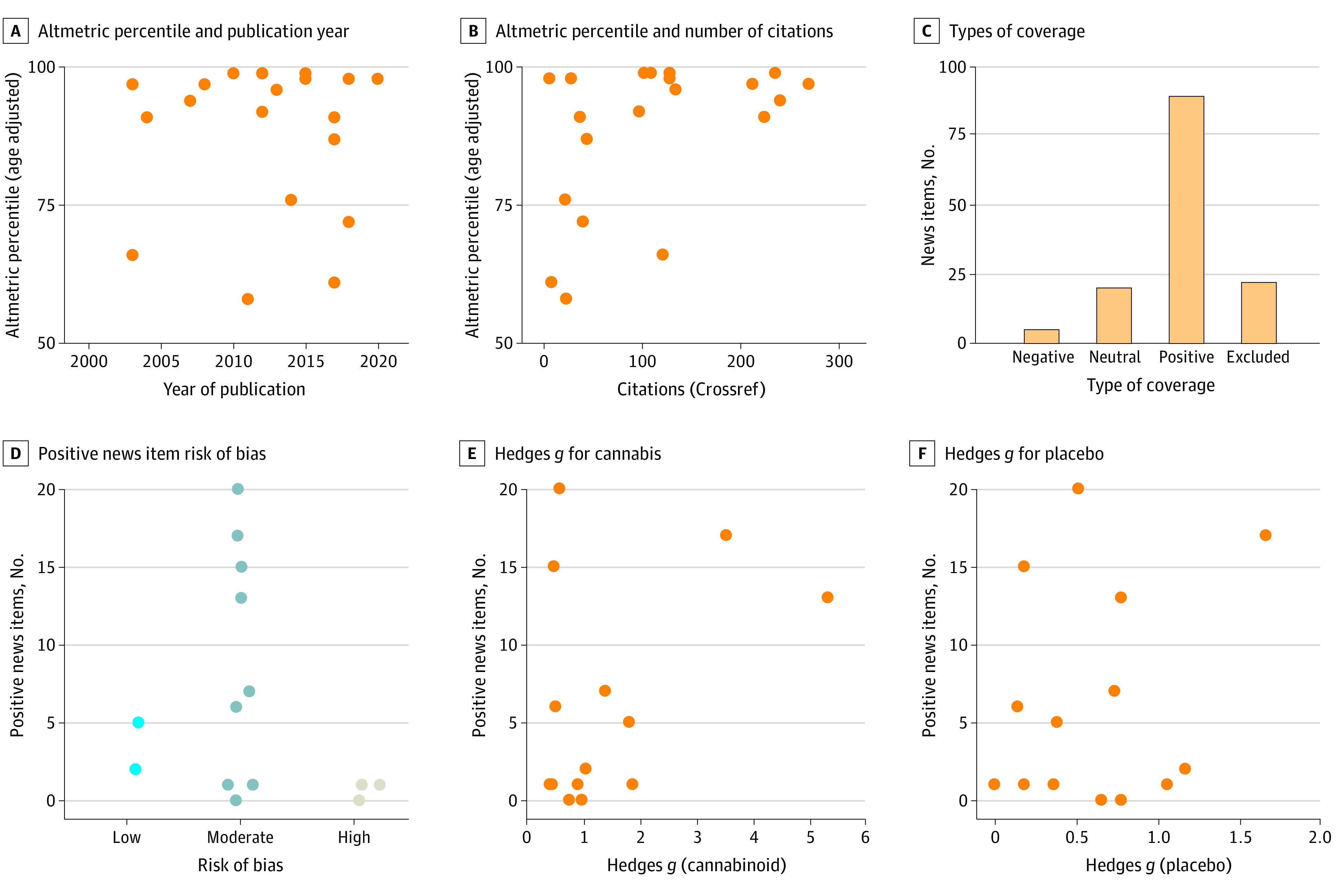
Association Between Media Attention and Effect Sizes of Individual Articles

We then investigated the academic and nonacademic impact of the articles in the meta-analysis. First, descriptively, the mean (SD) number of academic citations linked to each study in this meta-analysis was 125 (112). The studies were published in journals with a mean (SD) impact factor of 5 (2) (eTable 4 in Supplement 1). The mean (SD) Altmetric score, indicating the amount of nonacademic attention and engagement linked to each study, was 89 (115). Compared with all other scientific publications within the same 6-month period (age adjusted), the present studies on cannabinoid treatment for clinical pain were among the top 88% in terms of Altmetric scores (SD, 14%). The age-adjusted Altmetric measure showed no bias of year of publication because trials of cannabinoid therapy for pain had similarly high nonacademic impact during the last 2 decades (Figure 4A).

No significant correlation was found between the number of citations and the Altmetric percentile score (age adjusted) (ρ = 0.38, *P* = .10), indicating that these scores quantify different types of attention (Figure 4B). The Altmetric percentile score showed no significant correlation with the effect size of the placebo (ρ = 0.02, *P* = .95) or cannabinoid treatment (ρ = 0.25, *P* = .28). Likewise, academic citations showed no significant correlations for the effect size of the placebo *(ρ* = 0.09 *P* = .71) or cannabinoid treatment (ρ = 0.07, *P* = .76).

The raw Altmetric score compiles both academic sources (eg, attention by academics on Twitter) and wider media outlets, meaning they are slightly ambiguous for understanding how these articles have spread to the wider public. Furthermore, these scores do not indicate whether the attention these articles receive is positive or negative. To fix these issues and investigate the spread and type of attention these articles receive in popular media, we obtained the blog and news posts (collectively referred to as “news items”) associated with each article. Each news post was classified as positive, negative, or neutral about the outcomes of the cannabinoid treatment on pain (see Methods) (Figure 4C).

For the analysis of mass media impact, a total of 136 news items were included. Six of the 20 articles^14,18,19,22,25,31^ did not receive news items that matched the inclusion criteria. We saw no pattern between the number or proportion of positive news items and the study’s risk of bias or effect sizes. First, no clear patterns emerged relating to the positive news items and risk of bias (Figure 4D); however, there were relatively few low- and high-risk bias articles. Second, we saw no correlation between the number of positive news items and the outcome of the cannabinoid treatment (ρ = 0.22, *P* = .46) (Figure 4E) or placebo (ρ = 0.10, *P* = .74) (Figure 4F). Similarly, we saw no correlation between the percentage of positive news items an article received and the outcome of the cannabinoid treatment (ρ = 0.08, *P* = .77) or placebo (ρ = 0.11, *P* = .76).

Although these exploratory analyses into the association between article impact and influence with the effect size yielded no significant correlations, the result is meaningful. We can conclude that these articles receive considerable attention in the general media. Furthermore, this attention is independent of how biased the study is, how high the placebo response is, or how low the treatment effect is.

## Discussion

The data from the present meta-analysis, including 1459 patients with clinical pain, suggest that placebo responses contribute significantly to the pain reduction seen in cannabinoid randomized clinical trials. The size of the improvements in the placebo group was moderate to high and represents clinically relevant pain relief. In line with a recent meta-analysis^6^ that compared the superiority of cannabinoids vs placebo, our analysis did not yield a significant difference between genuine drug and placebo outcomes. The correlation between effect sizes in the placebo and drug group was high in the previous meta-analysis^6^ (*r* = 0.86), indicating that the 2 treatments share mechanisms of improvement, including spontaneous remission and placebo-like mechanisms of pain reduction.^7^

Our analysis of the association between each study’s risk of bias and the placebo effect size showed a significant result. Low risk of bias was linked to high placebo responses. Many placebo-controlled cannabinoid trials fail to ensure correct blinding, which is suggested to lead to an overestimation of the effectiveness of medical cannabis. In fact, many participants can distinguish between placebo and active cannabinoid, despite their having the same odor, taste, and appearance.^33^ It is possible that the trials with low risk of bias were successfully blinded and thus the participants in the placebo groups were more likely to maintain positive treatment expectations through the trial and benefit more from the placebo treatment. Because we had a particular interest in the blinding aspect of each study, we also provided a separate domain (in addition to the standard ROB2) that specifically reflects the blinding questions included in ROB2. Blinding per se was also significantly associated with placebo response, with successful blinding associated with higher placebo response. The blinding score was derived from ROB2 and was therefore not independent from the risk of bias assessment because they are derived from the same set of standardized questions.

There are numerous examples of the association between treatment expectations and placebo responses, even if recent theories suggest that placebo mechanisms extend beyond positive expectations. Instead, they can be seen as complex processes engaged by a variety of cues embedded in the rituals of medicine.^7^ In line with general principles of human perception,^34^ expectations of (possible) pain relief can modulate sensory processing and thereby reduce the perception of incoming nociceptive signals. Previous evidence^35^ also suggests that placebo analgesia may occur even in the presence of ambiguous or contradicting facts so that we create a percept that is tailored to our initial expectations.^36^ This finding may be crucial for understanding the lack of an association between trial duration and placebo effect size because the initial expectations when entering the trial should otherwise fade over time and yield lower placebo responses in trials with long duration. We did not find any significant difference in placebo response among the trials, irrespective of the duration, which ranged from 45 minutes to several months.

The unusually high attention and engagement linked to cannabinoid pain trials (indicated by age-adjusted Altmetric scores) was independent of the clinical results and may uphold high expectations and placebo responses in future trials. In particular, we found that news articles and blogs had a strong positive bias toward the efficacy of cannabinoids in pain therapy. The positive media attention on cannabinoids for pain relief could partly explain the placebo responses seen in this systematic review. Studies show that reports in the mass media and lay press and information obtained from the internet foster treatment expectations.^9^ The positive and extensive media attention may shape placebo responses in subsequent clinical trials, yet the current study is not powered to address this possibility. We therefore consider this question to be of high importance, as the positive reporting toward cannabinoids regardless of study quality and effect size may subsequently lead to increased expectations that may ultimately influence the outcomes in clinical trials, although more detailed study of this possible implication is needed. Placebo-controlled trials will continue to see large effect sizes in the placebo groups of properly blinded studies, and open-label trials will be heavily confounded by exaggerated media reports. Therefore, positive attention regardless of effect size or risk of bias could have far-reaching influence on clinical trials, regulatory decisions, clinical practice, and ultimately patient access to cannabinoids for pain relief.

There is always a risk that the blinding has been compromised in clinical trials. The risk can be even greater in trials on the efficacy of cannabinoids in which the psychotropic adverse effects may reveal the active substance. Cannabidiol is a nonpsychotropic agent that would be well suited for successful blinding. In our meta-analysis, only 3 of 20 articles^23,24,32^ used CBD as the active substance, which may have affected the overall result. Future trials should account for blinding when comparing different forms of cannabinoid treatments, because different forms may have differential blinding properties and related placebo responses.

### Limitations

This study has limitations. Because this meta-analysis combines trials with different settings, levels of quality, and lengths of follow-up, heterogeneity is to be expected.^37^ The data extracted for the meta-analysis had some considerable heterogeneity; therefore, the results need to be interpreted with caution. To address heterogeneity, we used a random-effects model, recommended by Borenstein et al.^38^ Post hoc analyses were not applied to the meta-analytical data. However, before the start of the study, our hypotheses and analyses were preregistered. We used a small number of moderators and did not implement post hoc analyses because we limited our search to a handful of predefined and independent comparisons.

## Conclusions

The findings of this systematic review and meta-analysis suggest that placebo responses contribute significantly to pain reduction in cannabinoid clinical trials. The unusually high media attention surrounding cannabinoid trials, with positive reports irrespective of scientific results, may uphold high expectations and shape placebo responses in future trials. This influence may impact the outcome of clinical trials, regulatory decisions, clinical practice, and ultimately patient access to cannabinoids for pain relief.

## Notes

Supplement 1. **eTable 1.** Search Strategies **eTable 2.** Excluded Articles and Reason for Exclusion **eTable 3.** Risk of Bias Assessments **eTable 4.** Each Trial’s Scientific Citations, Journal Impact Factor, and Altmetric Scores **eFigure.** Funnel Plot for the Risk of Publication Bias

Supplement 2.
Data Sharing Statement
