# Supplementary material for: Placebo Response and Media Attention in Randomized Clinical Trials Assessing Cannabis-Based Therapies for Pain: A Systematic Review and Meta-analysis
Source: JAMA Netw Open. 2022 Nov 28;5(11):e2243848. doi: 10.1001/jamanetworkopen.2022.43848 (PMC9706362; doi:10.1001/jamanetworkopen.2022.43848)
Supplement: Supplement 1. — eTable 1. Search Strategies eTable 2. Excluded Articles and Reason for Exclusion eTable 3. Risk of Bias Assessments eTable 4. Each Trial’s Scientific Citations, Journal Impact Factor, and Altmetric Scores eFigure. Funnel Plot for the Risk of Publication Bias [file jamanetwopen-e2243848-s001.pdf]

Supplementary Online Content

Gedin F, Blomé S, Pontén M, et al. Placebo response and media attention in randomized clinical trials assessing cannabis-based therapies for pain: a systematic review and meta-analysis. *JAMA Netw Open*. 2022;5(11):e2243848. doi:10.1001/jamanetworkopen.2022.43848

eTable 1. Search Strategies

eTable 2. Excluded Articles and Reason for Exclusion

eTable 3. Risk of Bias Assessments

eTable 4. Each Trial's Scientific Citations, Journal Impact Factor, and Altmetric Scores

eFigure. Funnel Plot for the Risk of Publication Bias

This supplementary material has been provided by the authors to give readers additional information about their work.

© 2022 Gedin F et al. *JAMA Network Open.*

eTable 1. Search Strategies

Medline (Ovid)

1	Cannabis/	8926
2	exp "Marijuana Use"/	5301
3	Medical Marijuana/	1116
4	exp Cannabinoids/	13758
5	(bhang* or cannabi* or ganja* or hash* or hemp* or marijuana* or marihuana* or marijuana).ti,ab,kf.	55503
6	or/1-5	60051
7	exp Pain/	389370
8	(pain* or neuralgia*).ti,ab,kf.	687785
9	or/7-8	831552
10	Randomized Controlled Trial.pt.	501769
11	Controlled Clinical Trial.pt.	93575
12	randomized.ab.	472899
13	placebo.ab.	205951
14	Clinical Trials as Topic.sh.	190378
15	randomly.ab.	329139
16	trial.ti.	214952
17	or/10-16	1275136
18	exp Animals/ not Humans.sh.	4678248
19	17 not 18	1173020
20	6 and 9 and 19	**424**

© 2022 Gedin F et al. *JAMA Network Open.*

Embase (embase.com)

('cannabis'/de OR 'cannabis use'/exp OR 'medical cannabis'/de OR 'cannabinoid'/exp OR bhang*:ti,ab,kw OR cannabi*:ti,ab,kw OR ganja*:ti,ab,kw OR hash*:ti,ab,kw OR hemp*:ti,ab,kw OR marijuana*:ti,ab,kw OR marihuana*:ti,ab,kw OR marijuana:ti,ab,kw)

AND

('pain'/exp OR pain*:ti,ab,kw OR neuralgia*:ti,ab,kw)

AND

('controlled clinical trial'/exp OR random*:ab OR placebo:ab OR 'clinical trial (topic)'/exp OR trial:ti) NOT ([animals]/lim NOT [humans]/lim)

NOT [medline]/lim **= 487**

Updated literature search

The updated literature search was performed in September of 2021. There were no modifications made to the literature search strategy and the purpose of the updated search was only to include newly published articles.

Embase (embase.com)

('cannabis'/de OR 'cannabis use'/exp OR 'medical cannabis'/de OR 'cannabinoid'/exp OR bhang*:ti,ab,kw OR cannabi*:ti,ab,kw OR ganja*:ti,ab,kw OR hash*:ti,ab,kw OR hemp*:ti,ab,kw OR marijuana*:ti,ab,kw OR marihuana*:ti,ab,kw OR marijuana:ti,ab,kw)

AND

('pain'/exp OR pain*:ti,ab,kw OR neuralgia*:ti,ab,kw)

AND

('controlled clinical trial'/exp OR random*:ab OR placebo:ab OR 'clinical trial (topic)'/exp OR trial:ti) NOT ([animals]/lim NOT [humans]/lim)

NOT [medline]/lim

AND [randomized controlled trial]/lim AND [2020-2021]/py

= 42

© 2022 Gedin F et al. *JAMA Network Open.*

Medline ovid

1	Cannabis/	7822
2	exp "Marijuana Use"/	6458
3	Medical Marijuana/	1449
4	exp Cannabinoids/	15548
5	(bhang* or cannabi* or ganja* or hash* or hemp* or marijuana* or marihuana* or marijuana).ti,ab,kf.	63608
6	or/1-5	67143
7	exp Pain/	418520
8	(pain* or neuralgia*).ti,ab,kf.	764157
9	or/7-8	913068
10	Randomized Controlled Trial.pt.	542796
11	Controlled Clinical Trial.pt.	94373
12	randomized.ab.	532993
13	placebo.ab.	221223
14	Clinical Trials as Topic.sh.	197233
15	randomly.ab.	365375
16	trial.ti.	247058
17	or/10-16	1392241
18	6 and 9 and 17	538
21	limit 18 to (humans and yr="2020 - 2021")	**56**

eTable 2. Excluded Articles and Reason for Exclusion

Publication Year	Author	Reason for exclusion				
		Population	Intervention	Control	Outcome	Other
2007	Abrams	HIV				
2019	Almog					Abstract
2009	Ambler					Abstract
2018	Amerongen	Healthy				
1974	Angelico					Letter
2019	Babalonis	Healthy				
2017	Babalonis	Healthy				
2014	Barbara					Abstract
2009	Chung					Abstract
2012	Cooper					Abstract
2017	Cooper					Abstract
2013	Cooper	Healthy				
2018	Cooper	Healthy				
2009	Ellis	HIV				
2007	Esfandyari	Healthy				
2016	Fionda					Abstract
1977	Gottschalk				Did not measure pain	
2004	Grotenhermen					Commentary
2018	Hunter					Abstract
2012	Kalliomaki	Healthy				
2013	Kalliomaki	Healthy				
2013	Kalliomaki	Healthy				
1975	Karnol				Did not measure pain	
2011	Klooker				Discomfort, not pain	
2008	Kraft	Healthy				
2014	Leocani					Abstract
2017	Levin	Not pain				
2013	Lötsch					Abstract
2017	Marinelli					Protocol paper
1975	Milstein	Healthy				
2003	Naef	Healthy				
2004	Naef	Healthy				
2009	Notcutt					Abstract

© 2022 Gedin F et al. *JAMA Network Open.*

2011	Ostenfeld,	Dentist patients				
2012	Pinsger					Abstract
2009/2010	Pittler					Commentary
2008	Redmond	Healthy				
2006	Roberts	Healthy				
2007	Rog			Uncontrolled follow-up		
2005	Salim					Secondary analysis of another article in study
2019	Schindler,					Abstract
2013	Sewell					Conference abstract
2009	Turcotte					Poster abstract
2011	Turcotte				Poster abstract	
2017	Turner					Conference abstract
2006	Wade					Follow-up study of Wade et al. (2004)
2013	Wallace					Abstract
2017	Wallace					Conference abstract
2019	Walter				Discrimination not intensity	
2010B	Ware			Active control		
2009	Wasan					Conference abstract
2018	Werth					Conference abstract
2019	Werth					Conference abstract
2016	Wilsey					Same participants as Wilsey et al. (2016)
2009	Zajick					Abstract
2015	Zalai					Abstract
2020	Covelli	No pain at baseline				

Articles that did not provide sufficient data	
Year	Author
2020	Abrams
2020	Almog
2017	Amerongen
2006	Beaulieu
2021	Bebee
2006	Blake
2008	Conte
2016	Cote

© 2022 Gedin F et al. *JAMA Network Open.*

2007	Hagenbach
1973	Hill
1978	Jochimsen
2010	Johnson
2003	Karst
2018	Lichtman
2014	Lynch
2020	Meuth
2008	Narang
1975	Noyes
2012	Portenoy
1977	Raft
2010	Selvarajah
2014	Serpell
1978	Staquet
2004	svendsen
2015	Turcott
2019	van de Donk
2004	Wade
2008	Wilsey
2013	Wilsey
2016	Wilsey,
2006	Wissel
2003	Zajicek
2008	Nietecka-Buchta

© 2022 Gedin F et al. *JAMA Network Open.*

eTable 3. Risk of Bias Assessments

The Risk-of-bias tool 2 (ROB 2) criteria were used with the addition of a sixth category for blinding.

Source	1. Bias arising from the randomization process	2. Bias due to deviations from intended interventions	3. Bias due to missing outcome data	4. Bias in measurement of the outcome	5. Bias in selection of the reported result	Bias due to blinding	**Overall risk of bias**
Berman, 2004 [12]	Low	Low	Low	Low	High	Low	**Moderate**
Buggy, 2003 [13]	Low	Low	Low	Low	High	Low	**Moderate**
Chaves, 2020 [14]	Low	Low	Low	Low	Low	Low	**Low**
Corey-Bloom, 2012 [15]	Low	Low	Moderate	Low	Low	Low	**Moderate**
De Vries, 2017 [16]	Low	Low	Moderate	Low	Moderate	Low	**Moderate**
Issa, 2014 [17]	Low	Low	Low	Low	High	Low	**Moderate**
Langford, 2013 [23]	Low	Low	Low	Low	Low	Low	**Low**
Malik,2017 [24]	Low	Low	Moderate	Low	Moderate	Low	**Moderate**
Nurmikko, 2007 [25]	Low	Low	Low	Low	Moderate	Low	**Moderate**
Rog, 2015 [22]	Low	Low	Low	Low	Moderate	Moderate	**Moderate**
Schimrigk, 2017 [18]	Low	Low	Low	Low	Moderate	Moderate	**Moderate**
Skrabek, 2008 [19]	Moderate	Moderate	Moderate	Moderate	High	Moderate	**High**
Toth 2012 [20]	Moderate	Moderate	Low	Low	High	Moderate	**High**
Turcott 2018 [21]	Moderate	High	High	Moderate	Moderate	High	**High**
Wade 2003 [27]	Moderate	Moderate	Low	Moderate	High	Moderate	**High**
Wallace, 2015 [26]	Low	Low	Low	Low	Low	Moderate	**Moderate**
Ware, 2010 [28]	Low	Low	Low	Low	Low	Moderate	**Moderate**
Weizman, 2018 [29]	Low	Moderate	Low	Low	Moderate	Moderate	**Moderate**
Zadikoff, 2011 [30]	Low	Moderate	Low	Low	Low	Moderate	**Moderate**
Zaijcek, 2012 [31]	Low	Low	Low	Low	Moderate	Moderate	**Moderate**

© 2022 Gedin F et al. *JAMA Network Open.*

eTable 4. Each Trial's Scientific Citations, Journal Impact Factor, and Altmetric Scores

Source	Crossref Academic citations	Journal Impact Factor	Altmetric score Non-academic citations	Altmetric Age adjusted percentile
Berman, 2004 [12]	219	6	20	91
Buggy, 2003 [13]	120	6	4	65
Chaves, 2020 [14]	5	3	169	99
Corey-Bloom, 2012 [15]	107	7	329	99
De Vries, 2017 [16]	37	5	26	91
Issa, 2014 [17]	22	3	5	76
Langford, 2013 [23]	129	5	33	96
Malik, 2017 [24]	23	3	3	61
Nurmikko, 2007 [25]	234	7	24	94
Rog, 2005 [22]	458	10	93	99
Schimrigk, 2017 [18]	41	6	15	87
Skrabek, 2008 [19]	208	6	42	97
Toth 2012 [20]	96	6	15	92
Turcott 2018 [21]	35	3	6	72
Wade 2003 [27]	263	3	59	97
Wallace, 2015 [26]	98	5	285	99
Ware, 2010 [28]	232	7	378	99
Weizman, 2018 [29]	25	8	212	98
Zadikoff, 2011 [30]	23	1	3	58
Zajicek, 2012 [31]	123	8	154	99
Mean (SD)	125 (112)	5 (2)	89 (115)	88 (14)

© 2022 Gedin F et al. *JAMA Network Open.*

eFigure 1. Funnel Plot for the Risk of Publication Bias

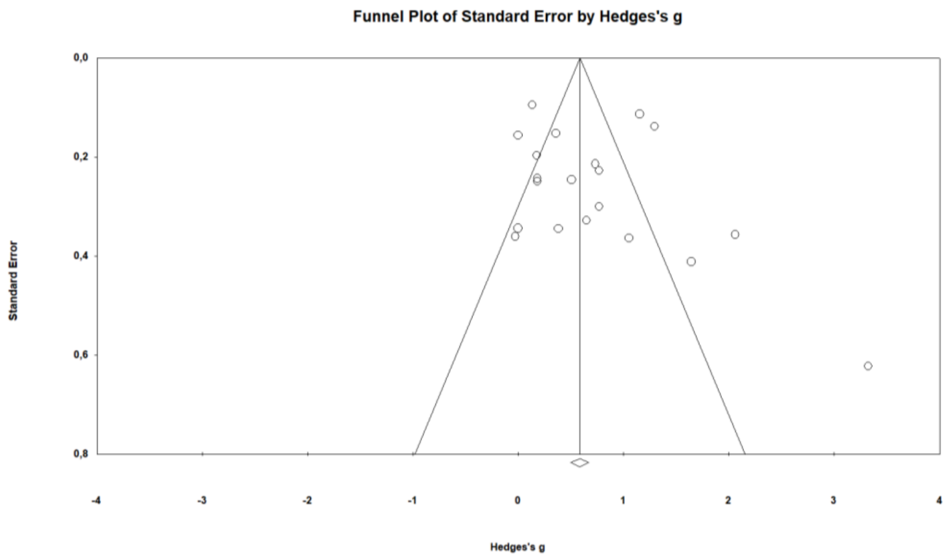

© 2022 Gedin F et al. *JAMA Network Open.*
