# Supplementary material for: Placebo Response and Media Attention in Randomized Clinical Trials Assessing Cannabis-Based Therapies for Pain: A Systematic Review and Meta-analysis
Source: JAMA Netw Open. 2022 Nov 28;5(11):e2243848. doi: 10.1001/jamanetworkopen.2022.43848 (PMC9706362; doi:10.1001/jamanetworkopen.2022.43848)
Supplement: Supplement 2. — Data Sharing Statement [file jamanetwopen-e2243848-s002.pdf]

Data Sharing Statement

Gedin. Placebo Response and Media Attention in Randomized Clinical Trials Assessing Cannabis-Based Therapies for Pain. *JAMA Netw Open*. Published November 28, 2022. doi:10.1001/jamanetworkopen.2022.43848

Data
Data available: No
